# Histopathological and Digital Morphometrical Evaluation of Uterine Leiomyoma in Brazilian Women

**DOI:** 10.1155/2016/2968410

**Published:** 2016-05-15

**Authors:** Ana Paula Fernandes da Silva, Luciano de Albuquerque Mello, Erlene Roberta Ribeiro dos Santos, Silvania Tavares Paz, Carmelita Lima Bezerra Cavalcanti, Mario Ribeiro de Melo-Junior

**Affiliations:** ^1^Programa de Pós-Graduação em Patologia, Universidade Federal de Pernambuco (UFPE), Campus Universitário, Avenida Morais Rego s/n, Cidade Universitária, 50670-910 Recife, PE, Brazil; ^2^Laboratório de Imunopatologia Keizo Asami (LIKA), UFPE, Recife, PE, Brazil; ^3^Centro Acadêmico de Vitória, UFPE, Vitória de Santo Antão, PE, Brazil; ^4^Departamento de Patologia, UFPE, Recife, PE, Brazil

## Abstract

The current study aims to evaluate histopathological and digital morphometrical aspects associated with uterine leiomyomas in one hundred and fifty (150) patients diagnosed with leiomyoma. Uterine tissues were subjected to the histopathological and digital morphometric analyses of the interstitial collagen distribution. The analysis of medical records indicates that most of the women diagnosed with uterine leiomyomas (68.7%) are between 37 and 48 years old. As for the anatomic location of the tumors, approximately 61.4% of the patients had intramural and subserosal lesions. In 50% of the studied cases, the patients developed uterine leiomyomatosis (with more than eight tumors). As for the morphometric study, the average size of the interstitial collagen distribution held approximately 28.53% of the capture area, whereas it was of 7.43% in the normal tissue adjacent to the tumor. Another important aspect observed in the current study was the high rate of young women subjected to total hysterectomy, a fact that resulted in early and definitive sterility.

## 1. Introduction

Uterine smooth muscle neoplasms comprehend benign tumors called leiomyomas, which are usually multiple and derived from myometrial smooth muscle cells, as well as malignant tumors, leiomyosarcomas, of low incidence, which develop from myometrium mesenchymal cells [1, 2].

Uterine leiomyoma is the most common benign neoplasia of the female genital tract. It affects 20–40% of women in reproductive age [3, 4]. This is a uterine body proliferative lesion derived from a mutagenic change that results in the loss of growth regulation mechanisms of the myometrium muscle cells [2]. Such lesion may be classified as subserosal, submucosal, or intramural leiomyoma, according to its anatomical location [4, 5].

The disease risk profile comprises black women with family history of uterine leiomyomas, aged between 35 and 39 years, showing low parity, infertility or tubal ligation, recurrent gynecological infection, and chronic diseases carriers, and with high body mass index (BMI) [1–4, 6].

The development of leiomyomas depends on a complex interaction among female gonadal steroid estrogen and progesterone hormones, growth factors, cytokines, genetic predisposition, and somatic mutations. Although its etiology remains unclear, it is known that the loss of the myomatous cell growth regulation occurs mainly by the suppression of apoptosis-inducing protein B-cell lymphoma 2 (bcl-2) and p27 [7–9].

The rapid leiomyoma growth may result in functional changes in organs adjacent to the tumor as well as in several problems to women's health. The most common clinical manifestations are increased and irregular menstrual flow, pain in the pelvic region, and increased abdominal size [10].

The diagnosis is easily accomplished by analyzing the symptoms and imaging exams that guide the therapeutic approach to be applied [2]. The histochemical techniques for evaluating the tumor histological pattern and the immunohistochemical study of tumor markers are essential to feature the leiomyoma type and the differential diagnosis of leiomyosarcoma [10–12].

The tissue microscopic features analyses are an important tool to qualify and quantify histopathological parameters that enable differential diagnosis of the neoplasm. Uterine leiomyomas histomorphological changes—subsequent to the increased angiogenesis rate and the proliferation rate of myomatous and fibroblast cells—usually result in homogeneous appearance hypertrophic tissue, without sharp cellular atypias or necrosis consisting of large bundles of smooth muscle cells, which are crisscrossed and arranged in fascicles mimicking the normal myometrium appearance. They may also display fibrotic areas, hyaline or mucoid degeneration, and dystrophic calcifications in the adjacent tissue [13–15].

Therefore, the current study aims to evaluate the histopathological and digital morphometrical aspects associated with uterine leiomyomas in patients treated in the public health service of Pernambuco state (Brazil) and diagnosed with leiomyomatous nodules treated with uterine body myomectomy or with total or partial hysterectomy.

## 2. Materials and Methods

### 2.1. Cases Selection

One hundred and fifty (150) paraffin blocks containing uterine tissue fragments from women previously diagnosed with leiomyoma were selected. The samples obtained in the files from the Pathology Department at the Keizo Asami Immunopathology Laboratory (LIKA), which is a supplementary service of the Federal University of Pernambuco (UFPE), derive from surgical specimens from patients aged between 25 and 87 years (average of 43.1 years), treated in 33 health facilities spread across all regions of Pernambuco state (Zona da Mata, Agreste and Sertão).

### 2.2. Histochemical Study

The slides for histological assembly were chemically cleaned and degreased with detergent solution. After cleaning, a thin layer of resin extracted from* Aloe vera* leaves was applied to the slides in order to ensure adherence and prevent tissue detachment during staining procedures.

After the paraffin blocks underwent microtomy, the histological cuts (5 *μ*m) were deparaffinized, hydrated, and subjected to Masson's trichrome (MT) staining technique to show the interstitial collagen deposition. Subsequently, the slides were dehydrated in 95% ethanol, diaphonized in xylene, and mounted with glass slide and Entellan®.

### 2.3. Digital Morphometric Analysis

As for the interstitial collagen analysis, three fields were selected in each case, in which images were captured with final magnification of 100x. The histomorphometric study of the images in the histological slides was performed by a workstation consisting of a CCBBW 410 (Samsung®) video camera system coupled to an optical microscope (Olympus BH-2) and to a computer containing the MOTIC Image Plus 2.0 software.

The adopted morphometric parameter was the interstitial collagen average size (in pixels) distribution per field captured in the histological slide (total area of the field = 12 234 *μ*m^2^) using GIMP software,* GNU Image Manipulation Program*, version 2.8.1. Normal myometrium areas selected from the leiomyoma margins in each case study were used as controls.

Data from the digital morphometric study were analyzed using paired Student's *t*-test with significance level of 5% (*p* < 0.05) by means of the GraphPad PRISM® 5.0 software.

## 3. Results

**Figure 1:**
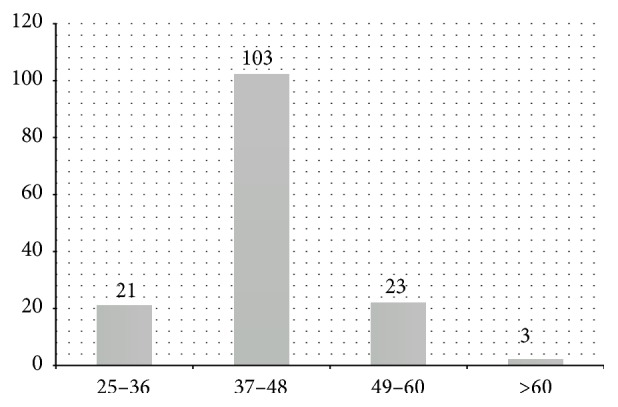
Age distribution of patients diagnosed with uterine leiomyoma in Pernambuco (Brazil) from 2001 to 2002.

Among the 150 cases analyzed in the current study, the most frequent ones (68.7%) were found in women aged between 37 and 48 years (Figure 1). This finding reinforces the hypothesis of the role played by hormonal dysregulation in the development of uterine body tumors. On the other hand, 82.7% of them (124 cases) were found in patients under the age of 48, in other words, patients who were in their reproductive age period at the time of the surgical treatment.

**Figure 2:**
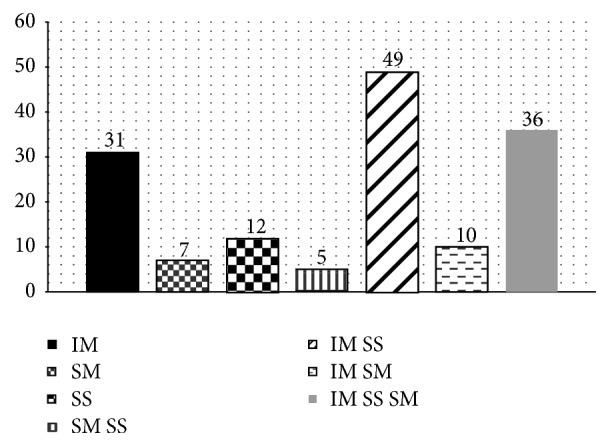
Leiomyomatous nodules distribution found in women from Pernambuco in the period from 2001 to 2002 according to their anatomical location: intramural (IM), submucosal (SM), subserosal (SS), submucosal and subserosal (SM SS), intramural and subserosal (IM SS), intramural and submucosal (IM SM), and intramural, subserosal, and submucosal (IM SS SM).

Another parameter evidenced in study was the leiomyomatous nodules distribution according to the anatomical classification (Figure 2). Thirty-two point seven percent (32.7%) of the patients had nodules located in the intramural and subserosal regions. In 24% of the cases, it was possible to visualize tumors distributed in all anatomical regions.

**Figure 3:**
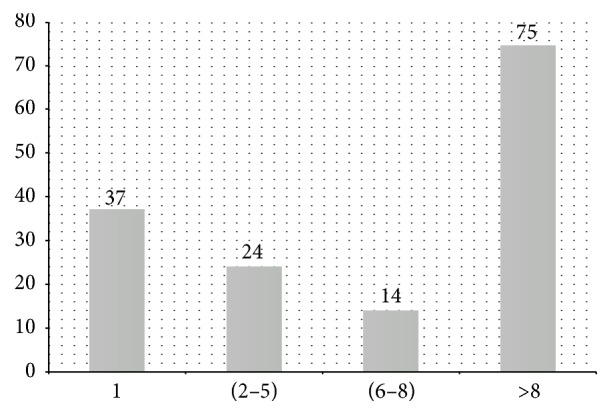
Frequency of the number of leiomyomatous nodules per patient in Pernambuco, Brazil.

In addition to the anatomic location, the amount of tumors per patient was also evaluated (Figure 3). Among the 150 patients, 75 showed more than eight histologically benign tumors in the uterine body, thus featuring uterine leiomyomatosis in 50% of the cases.

Regarding the type of surgical procedure, total hysterectomy was the most frequent one, and it was performed in 142 patients (94.7%); 54 of them were aged between 25 and 40 years, including 35 cases of single nodules with diameter smaller than 4 cm. Partial hysterectomy was performed in 5 patients (3.3%) and myomectomy was performed in 3 patients only (2%).

The histopathological analysis of tumor formations indicated the presence of vortexed muscle fibers or fibers in overlapping layers permeated by connective tissue with variable amount of blood vessels and fibroblasts immersed in an abundant extracellular matrix predominantly composed of collagen types I and III.

**Figure 4:**
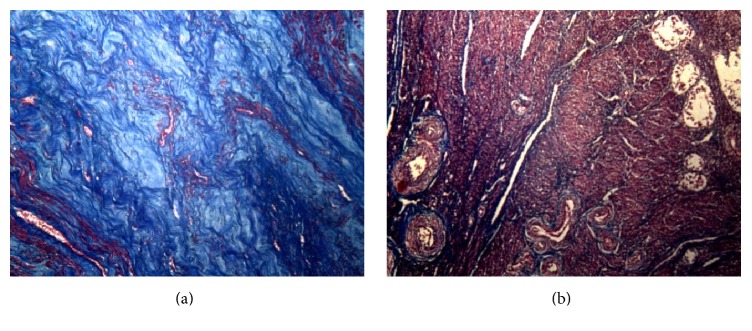
(a) Uterine leiomyoma. It is possible to see numerous collagen fibers of varied density and intensity (in blue). (b) Normal myometrium adjacent to the leiomyoma. Bundles of smooth muscle cells crisscrossed and arranged in fascicles (in red). Staining: Masson's trichrome. Magnification: 100x.

The photomicrographs of the leiomyomatous nodules central regions, which were taken using the same optical magnification, illumination, resolution, aperture, and camera shutter speed, show high density of collagen fibers with varying intensity. On the other hand, the normal myometrium in the leiomyoma margins shows the presence of discrete collagen fibers permeating muscle fibers (Figures 4(a) and 4(b)).

After cutting the areas of interest, the collagen shades (strong and weak blue) in the photomicrographs, the resulting image was subjected to histogram analysis in order to quantify the mean interstitial collagen area per captured field. The histogram study indicated that the mean collagen area in the leiomyomatous tumor regions was greater than that found in the normal myometrium at all morphometric analyses, with *p* < 0.0001 (Table 1).

## 4. Discussion

The prevalence of uterine leiomyoma reported in the current study met the estimates of other Brazilian epidemiological studies. Boclin and Faerstein [16], in a recent epidemiological study (2013), evaluated the demographic and socioeconomic features as well as the access to health services by the Brazilian population diagnosed with uterine myoma. They reported that 72% of the patients were younger than 45 years old. Their study also highlighted the 61.3% prevalence of uterine myoma diagnosis in the age group between 35 and 54 years. The age distribution results also corroborate recent American [3, 12, 17] and Portuguese [18] epidemiological findings.

The leiomyomas located in the uterine body are usually multiple and well differentiated. A single patient may show more than one type of nodule. Variations in the anatomical location directly interfere in the clinical manifestations, and the submucosal variant is often associated with infertility and metrorrhagia cases. In cases of intramural and/or subserosal location, the risk of circulatory disorders,* menorrhagic cycles*, and severe pain in the pelvic region is recurrent [15, 19].

Given the results on the surgical treatment performed in patients participating in the current study, the need to discuss the possibility of less invasive surgical alternatives such as endoscopic procedures, uterine artery embolization, or partial excisions that do not completely hamper the possibility of gestation in patients with uterine leiomyoma is imminent [20, 21].

The muscle fibers arrangement and the extracellular matrix patterns with collagen fibers predominance found in the current study correspond to the histomorphological pattern typical of most leiomyomatous nodules described in the literature [13, 14, 22–24].

Although the causes of leiomyoma remain unknown and its pathogenesis is still little explored in the literature, the primary mechanism suggested for the extracellular matrix increase in the uterine leiomyomas development location would be the* transforming growth factor beta* (TGF-*β*) protein increase, since this protein stimulates fibroblast proliferation, thus enhancing collagen production [15, 25].

## 5. Conclusions

According to the study results, it is concluded that the studied population showed higher frequency of uterine leiomyoma during the patients' reproductive age period, with higher prevalence of nodules located in the intramural and subserosal anatomical regions. In addition, 50% of the studied cases were featured as leiomyomatosis since the uteri showed eight or more tumors.

It is worth mentioning the fact that most patients were surgically treated with total hysterectomy, even in cases in which they had a single nodule with diameter smaller than 4 cm. This procedure led to the patients' total sterilization, although most of them were still in reproductive age.

In addition to the hypertrophy and hyperplasia of myometrial cells typical of the leiomyomatous neoplastic process, the histopathological study showed significant difference of the leiomyoma extracellular matrix in comparison to the normal myometrium, in all studied cases. The large amount of interstitial collagen found in the nodules analyzed in the current study shows its notorious role in the leiomyomatous tumor mass development. However, it should be emphasized that it is necessary to perform complementary studies in order to elucidate the mechanisms by which this fibroblast activity is intensified.

## Figures and Tables

**Table 1:** Interstitial collagen average area^*∗*^ per histological field in uterine tissue from women.

	Leiomyoma		Normal myometrium	
	Pixel	%	Pixel	%
Minimum	1201.00	3.90	479.00	1.60
Maximum	9128.00	70.00	6355.00	20.70
*Average*	*9442.66*	*28.53*	*2321.95*	*7.43*

^*∗*^Total area of the capture field = 12.234 μm^2^.
